# Financial incentives as a governmental tool to bridge the medical manpower gap between Israel’s center and periphery

**DOI:** 10.1186/s13584-019-0344-2

**Published:** 2019-10-14

**Authors:** Eyal Jacobson, Vered Ezra

**Affiliations:** 1 Dan-Petah Tikva District, Clalit Health Services, Petah Tikva, Israel; 2 Medical Directorate, Israel Ministry of Health, Yirmiyahu St 39, Jerusalem, Israel

**Keywords:** Periphery, Financial incentives, Physicians, Disparities

## Abstract

One of the major health disparities between Israel’s center and periphery relates to the physician to population ratio. To overcome it, the Israeli government launched a financial incentive program in 2011, in an attempt to encourage physicians to work in the periphery and in specialties experiencing major shortages. A recent IJHPR study found that residents who choose to work in a peripheral institution gave more weight to the grant in their decision-making process than did residents from central institutions. This finding lends support to the rationale behind the government program and suggests that it is an effective means of achieving the desired goal.

This commentary details how the program was repeatedly adjusted during the 2011–2018 period, in light of changing needs. As financial and human resources are expected to remain scarce in the future, the program must continue to be constantly evaluated and adjusted in order to maintain its effectiveness.

## Main text

Though it is a small country, significant health disparities exist between Israel’s geographic areas, as well as among its various socioeconomic and ethnic groups. These disparities are reflected in health indices such as life expectancy, infant mortality, the prevalence of diabetes and cancer, child obesity, smoking prevalence, and more. Health disparities also exist in, and are caused by, the availability of health services. The Israeli Ministry of Health (MOH) considers reducing healthcare disparities to be one of its major strategic goals. Each year, the MOH publishes a report summarizing the current situation and trends [1]. This report, in turn, serves as the basis on which the Ministry develops policies for reducing gaps in healthcare.

**Table 1:** Financial Incentive Program

Year	Specialties in need^1^		Other specialties	Total Budget (NIS)	# requests
	Center	Periphery	periphery		
	Grant (NIS)	Grant (NIS)	Grant (NIS)		
2011–2014	300 k	500 k	300 k	715,000 k	1569
2015	211 k	331 k	–	75,000 k	374
2016	130 k (resident) 120 k (fellow)	216 k (resident) 200 k (fellow)	500 k^2^ 216k^3^	75,000 k	210
2017	–	–	144k^4^ 288k^5^ 575k^6^	35,000 k	
2018–2019	150 k	250 k	Specialty required by the hospital director: 250 k^7^; 500 k^8^; 750 k^9^ Family medicine 250 k^10^; 500 k^11^ Psychiatric (center+periphery) 400k^12^; 300 k^13^; 150 k^14^	85,000 k	

^1^See text for detailed list of specialties in each year

^2^ Available for a limited number of specialists, who moved to peripheral hospitals

^3^Physicians who started residency in specialties in need as defined by the hospital director

^4^Psychiatric specialist, 5 years’ experience or less

^5^Physicians who started residency in Family medicine (periphery only), or Geriatrics at Shoham Hospital

^6^ Specialists who moved to peripheral hospitals

^7^ Physicians who started residency in specialties in need as defined by the hospital director

^8^Specialists (less than 10 years’ experience), in specialties in need as defined by the hospital director

^9^ Specialists (10 years’ experience or over), in specialties in need as defined by the hospital director

^10^Family medicine residency in clinics located in socioeconomic clusters levels 2–5

^11^ Family medicine residency in clinics located socioeconomic clusters level 1

^12^ Hospitals with low staffing shortage

^13^ Hospitals with moderate staffing shortage

^14^ Hospitals with high staffing shortage

The distribution of physicians among Israel’s major geographic areas is one of the parameters for which disparities are most significant. Physician prevalence represents a key factor in determining the availability of health services throughout the country. In 2011, the MOH, together with the Ministry of Finance, embarked on a financial incentive program to encourage physicians, residents, and specialists, to move to Israel’s geographic periphery. This program, which began as an agreement with the Israeli Medical Association (IMA), is still running and is frequently adjusted to meet current needs and maintain its efficacy (Table #1 summarizes the key elements of the financial incentive program since 2011).

In the original agreement with the IMA of 2011, it was stated that grants will be given to physicians who will start residency in specialties in need and in the periphery. The predefined cost framework was NIS 637.5 million for 8.5 years (2011 to 2019), NIS 75 million per year [2].

During the years 2011–2014, due to the deepening physician shortage and the accompanying public discourse, grants were given to all physicians starting residencies in the periphery (NIS 500 k for specialties in need and NIS 300 k for other specialties) as well as to physicians starting residencies in specialties in need in the center of Israel (NIS 300 k). Specialties in need were defined as: Geriatrics, Hemato-Oncology, Neonatology, Pathology, Rehabilitation, Emergency Medicine, Nuclear Medicine, Anesthesiology, Pediatric and Adult Psychiatry, Internal Medicine, Surgery and Intensive Care. It was agreed that any deviation from the cost framework will be offset in the coming years. As a result, by the end of 2014, 1874 grants with total value of NIS 715 million were approved. Therefore, the cost framework of NIS 637.5 million, which was planned for 2011–2019, was fully utilized between by the end of 2014. Nevertheless, the Ministry of Finance decided to approve an additional NIS 75 million for the program in 2015.

In 2015, after examining the program effects and current needs, it was decided to redefine the list of specialties in need as follows: Anesthesia, Neonatology, Pathology, Emergency Medicine, Geriatrics, Child Psychiatry, Adult Psychiatry, Rehabilitation, Pediatric Hemato-Oncology, Nuclear Medicine and Family Medicine. In this year, too, grants were given only to residents (and fellows). The size of the grant for a specialty in need was NIS 211 k in the center and NIS 331 k in the periphery. Even though the size of the grants were now significantly lower, the ratio between the grants in the periphery and center was kept similar.

In 2016, an additional amount of NIS 75 million was approved for the program. The program was again re-evaluated, and it was decided that there is a need to further strengthen the periphery by giving most of the grants to physicians in the periphery, and by including not only residents but also board-certified specialists who will move to peripheral hospitals, according to the following categories:

- Grants to specialists who will move to the periphery: NIS 500 k (total of about NIS 24 million).
- Grants to physicians who will start residency in the periphery in specialties that will be defined by each hospital director: NIS 216 k (total of about NIS 9.7 million).
- Grants to physicians who will start residency or fellowship (sub-specialty) in specialties in need (Geriatrics, Hemato-Oncology, Neonatology, Pathology, Rehabilitation, Nuclear Medicine, Pediatric Surgery, Pediatric Intensive Care, Forensic Medicine, and Radiotherapy):
  - NIS 216 k for a resident in the periphery (NIS 130 k in the center).
  - NIS 200 k for a fellow in the periphery (NIS 120 k in the center).

In 2017, an additional budget of NIS 35 million was allocated to the program. Due to the low budget and the current needs of the system, it was decided to focus only on the periphery as well as on the psychiatric specialty that was in a great need:

- Grant to specialists who move to the periphery: NIS 500 k (total of NIS 25 million).
- Grants for physicians starting family medicine residency in the periphery: NIS 250 k (total NIS 5 million).
- Grants for special training of psychiatrists in the periphery: NIS 125 k (total of NIS 1.5 million).
- Exceptions Committee – total budget of 3.5 NIS million.

In 2018 a budget of 70 NIS was allocated for the program for 1.5 years. Following a thorough re-evaluation of the program with hospital and HMO directors, the criteria for the grants were redefined as follows:

- Grants for physicians in the periphery in specialties that will be defined by each hospital directors. Each peripheral hospital received a budgetary framework: Specialist with 10 years or more of experience - NIS 750 k; Specialist with less than 10 years of experience - NIS 500 k; Resident - NIS 250 k (total of NIS 24 million).
- Grants for physicians who start residencies in specialties in need (Geriatrics, Hemato-Oncology, Neonatology, Pathology, Rehabilitation, Emergency Medicine, Nuclear Medicine, Anesthesiology, Psychiatry, Pediatric Surgery, Vascular Surgery, Pediatric Intensive Care, and Forensic Medicine): NIS 150 k and NIS 250 k in the center and in the periphery, respectively (Total NIS 26.7 million).
- Grants for residents in Family Medicine in the periphery according to the socioeconomic level of the place of clinic. Each HMO received a budgetary framework: NIS 500 k and NIS 250 k for socioeconomic clusters 1 and 2–5 respectively (total NIS 8.9 million).
- Grants for residents in Psychiatry in the center and the periphery according to the level of staffing shortage: NIS 400 k, NIS 300 k and NIS 150 k for high, medium and low staffing shortage, respectively (total NIS 6.8 million).
- Grants for specialists in Psychiatry in maximum security hospitals: NIS 750 k and NIS 500 k for a specialist with 10 or more years of experience and less than 10 years of experience, respectively (total NIS 1 million).
- Grants for residents in Public Health: NIS 150 k (total NIS 500 k).
- Grants for physicians who will move to work in the Tuberculosis Department at Shmuel Harofeh Hospital (total NIS 1.2 million): residents - NIS 400 k; general physicians - 250 NIS; specialists with less than 10 years of experience - NIS 500 k; specialists with 10 years or more of experience - NIS 750 k.

Wasserstrum et. Al. recently published an IJHPR article evaluating the ability of the monetary grants in Israel to influence physicians who started their residency between 2012 and 2014 [3]. Unsurprisingly, the researchers found that physicians who chose a remotely-located institution (RLI) as their place of residency attributed significantly more importance to the grant when making their decision compared to residents from centrally-located institutions (CLI). The effect remains significant in a multivariate model.

This finding is consistent with the Brookdale Institute research report dealing with the same issue, which found that a higher rate of physicians from RLIs claimed that financial incentives played a positive role in their decision of where to practice as compared to physicians from CLIs (50 and 30%, respectively) [4].

## Conclusions

These consistent findings support the rationale behind the government program aimed at encouraging physicians to move to the periphery of Israel and suggest that it is effective. As there are a limited number of newly licensed physicians each year, theoretically the success of the program over its first years could even eliminate the major shortages that existed in certain specialties while exacerbating or creating shortages in other specialties not covered in the incentive program. For that reason, as well as other reasons, the program, now in its eighth year, is being adapted to meet the evolving needs of the healthcare system. It is also becoming more flexible, as hospital directors have gradually gained more autonomy with regard to determining the criteria that influence which types of physicians are entitled to receive financial incentives.

As financial and human resources are expected to decrease even more in the future, the financial incentives allocated to this issue need to be optimized. The problem should be redefined periodically, and the solutions should be designed accordingly. Furthermore, as the ratio between licensed physicians and open positions for residency is expected to grow, the need for national programs such as the one studied should be re-considered.

The next collective agreement is expected to be signed in 2020. The impending decisions regarding financial grants present an opportunity to design studies that will prospectively assess the efficacy of financial incentives by collecting information on the attitudes of current medical students and comparing them with the decisions they make after the terms of the new agreement are revealed.

## Data Availability

Not applicable.

## Abbreviations

CLI: Centrally-located institution
IJHPR: Israel Journal of Health Policy Research
IMA: Israel Medical Association
MOH: Ministry of Health
NIS: New Israeli Shekel
RLI: Remotely-located institution
